# Inverse associations between serum urate and glycemic status in a general population and in persons with diabetes mellitus

**DOI:** 10.1186/s13098-020-00528-0

**Published:** 2020-03-10

**Authors:** Ichiro Wakabayashi

**Affiliations:** Department of Environmental and Preventive Medicine, Hyogo College of Medicine, Mukogawa-cho 1-1, Nishinomiya, Hyogo 663-8501 Japan

**Keywords:** Cardiovascular risk factor, Diabetes, Hemoglobin A1c, Metabolic syndrome, Urate

## Abstract

**Background:**

Results of previous studies regarding the relationship between blood urate level and diabetes are conflicting. The aim of this study was to clarify the relations of urate with glycemic status and metabolic syndrome in a general population and in patients with diabetes.

**Methods:**

The participants were 12,528 men receiving health-checkup examinations (29–70 years old) and they were divided into four quartile groups for serum urate levels. Relationships of urate with metabolic syndrome and its components were investigated after adjustment for age and lifestyle factors such as smoking, alcohol drinking and regular exercise.

**Results:**

In the overall participants and the participants with diabetes (n = 802), hemoglobin A1c levels were significantly lower in the 2nd, 3rd and 4th quartiles for urate than in the 1st quartile and the levels tended to be lower with an increase in the quartile. In the overall participants, the adjusted odds ratios for diabetes vs. the 1st quartile of urate were significantly lower in the 2nd quartile (0.71 [0.59–0.87])), 3rd quartile (0.51 [0.41–0.63]) and 4th quartile [0.35 (0.28–0.44)) than the reference level and the odds ratio tended to be lower with an increase in the quartile. A high urate level was positively associated with visceral obesity, hypertension, dyslipidemia (high triglycerides and/or low HDL cholesterol) and metabolic syndrome, and these associations were less prominent in the diabetes group than in the overall participants.

**Conclusions:**

Blood urate level is inversely associated with glycemic status in both a general population and patients with diabetes. Positive associations of urate with metabolic syndrome and its components except for diabetes are confounded in the presence of diabetes.

## Background

Hyperuricemia is a risk factor for atherosclerosis and is involved in the pathogenesis of cardiovascular disease [1]. There has been an accumulation of information on the associations of urate with metabolic syndrome and its components such as obesity, hypertension and dyslipidemia [2–4]. However, the relationship of urate with diabetes is still not clear. According to the results of a meta-analysis study using data for 8 prospective studies, a high serum urate level is associated with the risk of type 2 diabetes in middle-aged people and elderly people [5]. However, the results of a prospective study on men and women in US communities suggested that urate level increases prior to diagnosis of diabetes and then declines with the duration of diabetes [6, 7]. A bell-shaped relationship between glycemic status and serum urate level has been found in previous cross-sectional studies [8, 9]: Compared with a group of subjects with low glycemic levels, urate levels were high in a group of subjects with medium glycemic levels and were low in a group of subjects with high glycemic levels. In a biracial population of Fiji, compared with the levels in a normo-glycemic group, urate levels were high in a group with impaired glucose tolerance and were low in a group with diabetes [10]. These findings of the cross-sectional study agree with the aforementioned findings of the prospective study [6, 7]. On the other hand, in a previous cross-sectional study, serum urate level showed linear inverse associations with glycemic status and prevalence of diabetes [3]. Moreover, in a prospective study on healthy Korean men, fasting glucose was associated with incidental hyperuricemia [11]. Regarding patients with diabetes, there has been limited information on the relationship between urate and glycemic status. An inverse association between serum urate level and glycemic status was reported in Iranian patients with diabetes [12]. In a recent study in China, it was shown that serum urate level was inversely correlated with hemoglobin A1c in patients with type 2 diabetes but was positively correlated with hemoglobin A1c in normo-glycemic subjects [13]. Thus, conflicting results have been reported for the relationships of blood urate level with glycemic status in both a general population and patients with diabetes. However, there have been few studies in which the relationship between blood urate level and glycemic status was investigated simultaneously in a general population and in patients with diabetes using the same cohort.

The purpose of this study was therefore to clarify the relationship between serum urate level and glycemic status in middle-aged men in a general population and in patients with diabetes using results of health checkup examinations in a large cohort. Glycemic status was evaluated by hemoglobin A1c, and hemoglobin A1c and other components of metabolic syndrome were compared in quartile groups for serum urate level.

## Methods

### Subjects

The subjects were Japanese men aged 29–70 years (n = 12,528) who had received periodic health checkup examinations at workplaces in Yamagata Prefecture in Japan. The original database used in this study was a collection of the results of annual health checkup examinations performed from April 2005 to March 2006 for workers in a district of Japan. The database used in this study was supplied by a health checkup institute, by which the questionnaires were prepared. Informed consent was not obtained from each subject, and the protocol of this study including no informed consent from each subject was approved by the Ethics Committee of Yamagata University School of Medicine (No. 112 from April 2005 to March 2006, approved on March 13, 2006) and the Hyogo College of Medicine Ethics Committee (No. 3003 in 2018).

Histories of alcohol consumption, cigarette smoking, regular exercise (almost every day for 30 min or longer per day), illness, and therapy for illness were self-reported by questionnaires. The questionnaires used in this study were prepared by the health checkup institute and include standard questions on individual lifestyles and histories of disease and medication therapy. Names of drugs used for treatment of each disease and information on Japanese traditional medicines used for therapy for diabetes and hyperuricemia were not included in the questionnaire used in this study. Since most people do not have a good understanding of the names of drugs they are taking, history of medication therapy was simply asked in the questionnaire for each of the diseases including hypertension, dyslipidemia, hyperuricemia and diabetes as “Are you receiving medication therapy to lower blood pressure level?”, “Are you receiving medication therapy to lower blood cholesterol level?”, “Are you receiving medication therapy to lower blood urate level?”, and “Are you receiving insulin injection or medication therapy to lower blood sugar level?”, respectively.

Individuals for whom information on any of the variables tested in this study was lacking were excluded from the subjects of this study. Individuals receiving medication therapy for hyperuricemia were also excluded from the subjects of this study. A cross-sectional study was performed using a local population-based database for the above subjects.

Average alcohol consumption of each subject per week was reported on questionnaires. Frequency of habitual alcohol drinking was asked in the questionnaire as “How frequently do you drink alcohol?”. Frequency of weekly alcohol drinking was categorized as “every day” (regular drinkers), “sometimes” (occasional drinkers) and “never” (nondrinkers). Cigarette smokers were defined as subjects who had smoked for 6 months or longer and had smoked for the past month or longer. Then the subjects who were smokers were further asked “What is your average cigarette consumption per day?”. The response categories for this question were “less than 21 cigarettes per day”, “21 or more and less than 41 cigarettes per day” and “41 or more cigarettes per day”.

### Measurements

Height and body weight were measured with the subjects wearing light clothes at the health checkup. Body mass index (BMI) was calculated as weight in kilograms divided by the square of height in meters. Waist circumference was measured at the navel level according to the recommendation of the definition of the Japanese Committee for the Diagnostic Criteria of Metabolic Syndrome [14], and visceral obesity was evaluated by the ratio of waist circumference (cm) to height (cm) (waist-to-height ratio: WHtR). Blood pressure was measured by trained nurses, who were part of the local health-checkup company, with a mercury sphygmomanometer once on the day of the health checkup after each subject had rested quietly in a sitting position. Korotkoff phase V was used to define diastolic pressure. Fasted blood was sampled from each subject in the morning, and serum urate, triglycerides and HDL cholesterol were measured by enzymatic methods using commercial kits, pureauto S UA, pureauto S TG-N and cholestest N-HDL (Sekisui Medical Co., Ltd, Tokyo, Japan), respectively. Hemoglobin A1c was measured by NGSP (the National Glycohemoglobin Standardization Program)-approved technique using the latex cohesion method with a commercial kit (Determiner HbA1c, Kyowa Medex, Tokyo, Japan). Since the standards of hemoglobin A1c used for measurement are different in the NGSP method and JDS (the Japan Diabetes Society) method, hemoglobin A1c values were calibrated by using a formula proposed by JDS [15]: hemoglobin A1c (NGSP) (%) = 1.02 × hemoglobin A1c (JDS) (%) + 0.25%. Coefficients of variation for reproducibility of each measurement were ≤ 3% for urate, ≤ 3% for triglycerides, ≤ 5% for HDL cholesterol and ≤ 5% for hemoglobin A1c.

### Criteria of metabolic syndrome

Metabolic syndrome was defined, according to the criteria by IDF (the International Diabetes Federation) [16] with a slight modification, as the presence of 2 or more risk factors in addition to visceral obesity diagnosed as large waist circumstance. Risk factors included in the criteria are visceral obesity, high blood pressure, dyslipidemina (low HDL cholesterol and/or high triglycerides) and hyperglycemia evaluated by hemoglobin A1c. The criterion for each risk factor was defined as follows: visceral obesity, WHtR ≥ 0.5; hypertension, systolic blood pressure ≥ 140 mmHg and/or diastolic blood pressure ≥ 90 mmHg; low HDL cholesterol, HDL cholesterol < 40 mg/dl; high triglycerides, triglycerides ≥ 150 mg/dl; diabetes, hemoglobin A1c ≥ 6.5%. Subjects receiving drug therapy for hypertension, dyslipidemia and diabetes were also included in the above definitions of hypertension, dyslipidemia and diabetes, respectively.

### Statistical analysis

The values of urate in participants were arranged in ascending order, and then the participants were divided into four quartile groups, and each variable was compared among the quartile groups for urate by univariate and multivariate analyses as explained below. Serum urate concentrations were measured as a unit of mg/dl with one decimal place, and there was a considerable number of subjects who showed the same value of urate concentration at each quartile border. Thus, it was impossible to divide the subjects into 4 quartile groups consisting of completely equal numbers of subjects. The subjects were therefore divided into 4 quartile groups with the similar numbers of subjects: 3150 subjects in the 1st quartile, 3214 subjects in the 2nd quartile, 2986 subjects in the 3rd quartile and 3178 subjects in the 4th quartile. Categorical variables were compared by means of Pearson’s Chi square test for independence. In univariate analysis, means of each variable were compared among the quartile groups by using analysis of variance (ANOVA) followed by Scheffé’s F-test as a post hoc test. In multivariate analysis, mean levels of each variable were compared by using analysis of covariance (ANCOVA) followed by Student’s t-test after Bonferroni correction. Triglyceride levels are known not to show a normal distribution. In fact, in the present study, triglyceride levels did not show a normal distribution (data not shown). They are therefore presented as a median with 25 and 75 percentile values and were compared among the groups non-parametrically by using Kruskal–Wallis test followed by Steel–Dwass test as a post hoc test in univariate analysis or were used after log-transformation in multivariate analysis. In logistic regression analysis, crude and adjusted odds ratios for metabolic syndrome and each of its components were estimated. Pearson’s correlation coefficients and standardized partial regression coefficients were calculated in univariate and multivariate linear regression analyses, respectively. Age, smoking, alcohol drinking and regular exercise were used as other explanatory variables or covariates in multivariate analyses. BMI was also added to the explanatory variables in analyses of variables other than WHtR and metabolic syndrome. In addition, histories of drug therapy for hypertension, dyslipidemia, and diabetes were adjusted for calculation of means of systolic or diastolic blood pressure, HDL cholesterol or log-transformed triglycerides, and hemoglobin A1c, respectively, in ANCOVA. Covariates were continuously or sequentially adjusted in multivariate analyses. Age and lifestyle-related factors including smoking, alcohol drinking and regular exercise are known to be possible confounders for the above relationships and thus were used as potential covariates in multivariate analyses. In addition, histories of medication therapy for diabetes, hypertension and dyslipidemia were used as covariates in analysis of hemoglobin A1c, blood pressure, and blood lipids, respectively, since each therapy directly influences the levels of these variables. Probability (*p*) values less than 0.05 were defined as significant. Statistical analyses were performed using a computer software program (SPSS version 16.0 J for Windows, Chicago IL, USA).

## Results

### Characteristics of the overall participants and those divided into quartile groups for urate level

The profile of the quartile groups of the participants for urate is shown in Table 1. Age was significantly younger in the 2nd, 3rd and 4th quartile groups than in the 1st quartile group. In the overall participants, the proportions of nondrinkers, regular drinkers and occasional drinkers were 20.1%, 45.4% and 34.5%, respectively. The proportions of subjects in the smoker categories were 44.5% (nonsmokers), 39.5% (less than 21 cigarettes per day), 15.5% (21 or more and less than 41 cigarettes per day) and 0.5% (41 or more cigarettes per day). The proportions of smokers were significantly smaller in the 3rd and 4th quartile groups than in the 1st quartile group, while the proportions of regular drinkers were significantly larger in the 2nd, 3rd and 4th quartile groups than in the 1st quartile group and the proportion tended to be larger with an increase in the quartile. Height, weight, waist circumference and BMI were significantly larger in the 2nd, 3rd and 4th quartile groups than in the 1st quartile group and tended to be larger with an increase in the quartile. When compared with the 1st quartile group for urate, the proportions of participants with a history of medication therapy for hypertension were significantly larger in the 3rd and 4th quartile groups. On the other hand, the proportions of participants with a history of medication therapy for diabetes were significantly smaller in the 3rd and 4th quartile groups than in the 1st quartile group and the proportion tended to be smaller with an increase in the quartile. The proportions of participants with hypertension, hyper-triglyceridemia, hypo-HDL-cholesterolemia, dyslipidemia and metabolic syndrome were significantly larger in the 3rd and 4th quartile groups than in the 1st quartile group, while the proportions of participants with diabetes were significantly smaller in the 2nd, 3rd and 4th quartile groups than in the 1st quartile group and the proportion tended to be smaller with an increase in the quartile.

**Table 1:** Characteristics of overall participants and participants in each quartile group for urate

	1st quartile Urate: 0.3–5.1 mg/dl	2nd quartile Urate: 5.2–5.9 mg/dl	3rd quartile Urate: 6.0–6.7 mg/dl	4th quartile Urate: 6.8–12.9 mg/dl	Overall Urate: 0.3–12.9 mg/dl
Number	3150	3214	2986	3178	12,528
Urate (mg/dl)	4.38 ± 0.68	5.56 ± 0.23**	6.33 ± 0.22**	7.59 ± 0.76**	5.96 ± 1.29
Age (years)	48.3 ± 9.5	47.1 ± 9.6**	46.4 ± 9.3**	46.6 ± 9.2**	47.1 ± 9.4
Smokers (%)	57.7	57.7	53.5**	53.5**	55.6
Drinkers (%)					
Occasional	35.1	34.3	35.5	33.2	34.5
Regular	38.9	43.1**	46.8**	52.9**	45.4
Regular exercise (%)	11.0	11.5	10.9	12.0	11.4
Height (cm)	169.3 ± 6.4	169.9 ± 6.1**	170.2 ± 6.2**	170.2 ± 6.1**	169.9 ± 6.2
Body weight (kg)	64.6 ± 10.1	66.8 ± 9.9**	68.8 ± 10.3**	71.4 ± 11.5**	67.9 ± 10.8
Waist circumference (cm)	80.4 ± 8.7	82.1 ± 8.6**	83.8 ± 8.3**	86.3 ± 8.9**	83.1 ± 8.9
BMI (kg/m^2^)	22.5 ± 3.1	23.1 ± 3.1**	23.7 ± 3.1**	24.6 ± 3.5**	23.5 ± 3.3
Triglycerides (mg/dl)	90 (62, 130)	95 (66, 141)	107 (73, 162)	130 (86, 204)	104 (70, 158)
Therapy for hypertension (%)	10.0	10.5	12.1**	15.1**	11.9
Therapy for dyslipidemia (%)	4.3	4.7	4.8	5.0	4.7
Therapy for diabetes (%)	5.2	4.4	3.4**	2.6**	3.9
High WHtR (%)	30.2	35.7**	43.1**	54.7**	40.9
Hypertension (%)	23.3	24.5	29.4**	38.4**	28.9
Hyper-triglyceridemia (%)	18.8	21.8**	29.5**	41.9**	28.0
Hypo-HDL cholesterolemia (%)	6.5	9.0**	10.0**	12.0**	9.3
Dyslipidemia (%)	24.4	28.6**	35.5**	46.9**	33.8
Diabetes (%)	8.9	6.8**	5.4**	4.5**	6.4
Metabolic syndrome (%)	8.4	8.9	10.7**	16.1**	11.0

Numbers and means with standard deviations, medians with interquartile ranges and frequencies (%) of each variable are shown

Asterisks denote significant differences from the 1st quartile group (** *p* < 0.01)

### Comparison of mean levels of each component of metabolic syndrome in the quartile groups for urate in overall participants

Means of each component of metabolic syndrome in the quartile groups of the overall participants for urate are shown in Table 2. WHtR and log-transformed triglycerides were significantly higher in the 2nd, 3rd and 4th quartile groups than in the 1st quartile group and tended to be higher with an increase in the quartile. Blood pressures (both systolic and diastolic) and HDL cholesterol were significantly higher and lower, respectively, in the 3rd and 4th quartile groups than in the 1st quartile group and tended to be higher and lower, respectively, with an increase in the quartile. Hemoglobin A1c was significantly lower in the 2nd, 3rd and 4th quartile groups than in the 1st quartile group and tended to be lower with an increase in the quartile [means and standard deviations (%) in univariate analysis: 5.52 ± 0.89 (1st quartile); 5.44 ± 0.69 (2nd quartile); 5.41 ± 0.60 (3rd quartile); 5.42 ± 0.57 (4th quartile)].

**Table 2:** Comparison of means of each variable in quartile groups for urate levels in overall participants

	1st quartile Urate: 0.3–5.1 mg/dl	2nd quartile Urate: 5.2–5.9 mg/dl	3rd quartile Urate: 6.0–6.7 mg/dl	4th quartile Urate: 6.8–12.9 mg/dl
WHtR				
Univariate	0.476 (0.474–0.477)	0.484 (0.482–0.485)**	0.493 (0.491–0.495)**	0.507 (0.506–0.509)**
Multivariate	0.473 (0.472–0.475)	0.483 (0.482–0.485)**	0.494 (0.492–0.496)**	0.509 (0.507–0.510)**
Systolic blood pressure (mmHg)				
Univariate	124.0 (123.4–124.5)	124.9 (124.4–125.4)	127.0 (126.4–127.6)**	130.1 (129.5–130.6)**
Multivariate	125.4 (124.8–125.9)	125.6 (125.1–126.1)	126.8 (126.2–127.3)**	128.2 (127.7–128.7)**
Diastolic blood pressure (mmHg)				
Univariate	75.4 (75.1–75.8)	76.3 (76.0–76.7)*	78.1 (77.7–78.5)**	80.6 (80.2–81.0)**
Multivariate	76.5 (76.2–76.9)	76.9 (76.5–77.2)	77.8 (77.5–78.2)**	79.2 (78.8–79.6)**
log (triglycerides [mg/dl])				
Univariate	1.97 (1.96–1.98)	2.00 (1.99–2.01)**	2.05 (2.04–2.06)**	2.14 (2.13–2.15)**
Multivariate	1.99 (1.98–2.00)	2.01 (2.00–2.02)*	2.05 (2.04–2.05)**	2.11 (2.10–2.12)**
HDL cholesterol (mg/dl)				
Univariate	59.1 (58.6–59.6)	57.0 (56.5–57.5)**	56.1 (55.6–56.6)**	54.9 (54.4–55.4)**
Multivariate	58.3 (57.8–58.8)	56.7 (56.2–57.1)**	56.2 (55.8–56.7)**	55.9 (55.4–56.3)**
Hemoglobin A1c (%)				
Univariate	5.52 (5.49–5.55)	5.44 (5.42–5.46)**	5.41 (5.39–5.44)**	5.42 (5.40–5.44)**
Multivariate	5.52 (5.50–5.54)	5.44 (5.42–5.46)**	5.42 (5.40–5.45)**	5.41 (5.38–5.43)**

Means with their 95% confidence intervals for each variable are shown. The range of urate levels in each quartile group is shown in the table. In multivariate analysis, adjusted odds ratios were estimated using age and histories of smoking, alcohol drinking and regular exercise as other explanatory variables. BMI was also adjusted in analysis of variables except for WHtR. In addition, a history of medication therapy for hypertension, dyslipidemia or diabetes was used as an explanatory variable in multivariate analysis for systolic and diastolic blood pressure, triglycerides, HDL cholesterol, and hemoglobin A1c

Asterisks denote significant differences from the 1st quartile (* *p* < 0.05; ** *p* < 0.01)

### Comparison of frequencies of metabolic syndrome and each component of metabolic syndrome in the quartile groups for urate in overall participants

Table 3 shows crude and adjusted odds ratios for metabolic syndrome and each risk factor in the 2nd, 3rd and 4th quartile groups for urate vs. the 1st quartile group in the overall participants. The odds ratios of the 3rd and 4th quartile groups vs. the 1st quartile group for metabolic syndrome, high WHtR, hypertension, hyper-triglyceridemia and hypo-HDL cholesterolemia were significantly higher than the reference level and tended to be higher with an increase in the quartile. The odds ratios of the 2nd quartile group for high WHtR and hypo-HDL cholesterolemia were also significantly higher than the reference level. The odds ratios for diabetes of the 2nd, 3rd and 4th quartile groups vs. the 1st quartile group [2nd quartile, 0.71 (0.59–0.87); 3rd quartile, 0.51 (0.41–0.63); 4th quartile, 0.35 (0.28–0.44)] were significantly lower than the reference level and tended to be lower with an increase in the quartile.

**Table 3:** Odds ratios for metabolic syndrome and its components in each quartile group vs. the 1st quartile group for urate in overall participants

	1st quartile Urate: 0.3–5.1 mg/dl	2nd quartile Urate: 5.2–5.9 mg/dl	3rd quartile Urate: 6.0–6.7 mg/dl	4th quartile Urate: 6.8–12.9 mg/dl
Metabolic syndrome				
Crude	1.00	1.06 (0.89–1.27)	1.30 (1.10–1.55)**	2.10 (1.79–2.45)**
Adjusted	1.00	1.16 (0.97–1.39)	1.51 (1.26–1.80)**	2.37 (2.01–2.79)**
High WHtR				
Crude	1.00	1.29 (1.16–1.43)**	1.75 (1.58–1.95)**	2.80 (2.52–3.10)**
Adjusted	1.00	1.42 (1.27–1.58)**	2.03 (1.82–2.27)**	3.36 (3.01–3.75)**
Hypertension				
Crude	1.00	1.07 (0.95–1.20)	1.37 (1.22–1.53)**	2.05 (1.83–2.28)**
Adjusted	1.00	1.03 (0.90–1.17)	1.26 (1.10–1.43)**	1.67 (1.47–1.90)**
Hyper-triglyceridemia				
Crude	1.00	1.21 (1.07–1.37)**	1.81 (1.61–2.04)**	3.12 (2.79–3.50)**
Adjusted	1.00	1.10 (0.97–1.25)	1.54 (1.36–1.75)**	2.45 (2.17–2.77)**
Hypo-HDL cholesterolemia				
Crude	1.00	1.41 (1.17–1.70)**	1.59 (1.32–1.92)**	1.95 (1.63–2.33)**
Adjusted	1.00	1.40 (1.15–1.70)**	1.55 (1.27–1.89)**	1.72 (1.41–2.09)**
Diabetes				
Crude	1.00	0.75 (0,62–0.90)**	0.58 (0.48–0.71)**	0.48 (0.39–0.60)**
Adjusted	1.00	0.71 (0.59–0.87)**	0.51 (0.41–0.63)**	0.35 (0.28–0.44)**

Odds ratios with their 95% confidence intervals for each variable are shown. The range of urate levels in each quartile group is shown in the table. In multivariate analysis, adjusted odds ratios were estimated using age and histories of smoking, alcohol drinking and regular exercise as other explanatory variables. BMI was also adjusted in analysis of variables except for high WHtR. In addition, a history of medication therapy for hypertension, dyslipidemia or diabetes was used as an explanatory variable in multivariate analysis for hypertension, hyper-triglyceridemia, hypo-HDL cholesterolemia and diabetes

Asterisks denote significant differences from the reference level of 1.00 (** *p* < 0.01)

### Comparison of frequencies of metabolic syndrome in the quartile groups for urate in the participants without diabetes

The relationship between urate and metabolic syndrome in participants without diabetes was also investigated by using logistic regression analysis. The crude odds ratios for metabolic syndrome of 2nd, 3rd and 4th quartiles vs. 1st quartile for urate were significantly higher than the reference level and tended to be higher with an increase in the quartile [2nd quartile, 1.27 (1.10–1.60), *p* < 0.05; 3rd quartile, 2.10 (1.70–2.59), *p* < 0.01; 4th quartile, 3.58 (2.93–4.38), *p* < 0.01]. Similar results were obtained in the multivariate analysis with adjustment for age and histories of smoking, alcohol drinking and regular exercise [2nd quartile, 1.35 (1.07–1.71), *p* < 0.05; 3rd quartile, 2.36 (1.90–2.93), *p* < 0.01; 4th quartile, 3.91 (3.17–4.81), *p* < 0.01].

### Characteristics of the participants with diabetes and those divided into quartile groups for urate level

Table 4 shows the profile of quartile groups for urate in the participants with diabetes. In the 4th quartile for urate, age was significantly older, body weight and waist circumstance were significantly larger, and BMI and triglycerides were significantly higher than in the 1st quartile. The percentage of participants receiving medication therapy for hypertension was significantly higher and the percentages of participants with hypertension, hyper-triglyceridemia, hypo-HDL cholesterolemia and dyslipidemia were significantly higher in the 4th quartile for urate than in its 1st quartile.

**Table 4:** Characteristics of the participants with diabetes and those in each quartile group for urate

	1st quartile Urate: 0.8–4.7 mg/dl	2nd quartile Urate: 4.8–5.5 mg/dl	3rd quartile Urate: 5.6–6.4 mg/dl	4th quartile Urate: 6.5–12.9 mg/dl	Overall Urate: 0.8–12.9 mg/dl
Number	190	202	224	186	802
Urate (mg/dl)	3.90 ± 0.73	5.17 ± 0.23**	6.00 ± 0.24**	7.31 ± 0.83**	5.59 ± 1.33
Age (years)	54.2 ± 7.8	53.6 ± 7.7	53.2 ± 7.9	51.7 ± 8.1*	53.2 ± 7.9
Smokers (%)	47.4	51.5	52.9	49.5	50.4
Drinkers (%)					
Occasional	33.2	37.6	37.9	39.8	37.2
Regular	38.4	36.1	42.4	43.0	40.0
Regular exercise (%)	19.5	13.9	12.9	15.1	15.2
Height (cm)	168.6 ± 6.6	168.1 ± 6.3	168.4 ± 5.9	169.4 ± 6.3	168.6 ± 6.3
Body weight (kg)	70.3 ± 12.4	72.1 ± 14.0	72.1 ± 12.6	75.8 ± 13.6**	72.5 ± 13.3
Waist circumference (cm)	86.7 ± 9.2	88.0 ± 10.7	87.9 ± 9.7	90.3 ± 9.9**	88.2 ± 9.9
Body mass index (kg/m^2^)	24.6 ± 3.6	25.4 ± 4.4	25.3 ± 3.8	26.3 ± 4.1**	25.4 ± 4.0
Triglycerides (mg/dl)	99 (65, 165)	122 (76, 190)	127 (84, 209)	137 (94, 219)**	122 (79, 194)
Therapy for hypertension (%)	24.7	35.1*	33.5	40.3**	33.4
Therapy for dyslipidemia (%)	14.2	19.8	17.4	21.0	18.1
Therapy for diabetes (%)	58.4	62.9	62.1	61.3	61.2
High WHtR (%)	62.6	64.9	66.1	69.4	65.7
Hypertension (%)	46.8	54.5	53.6	63.4**	54.5
Hyper-triglyceridemia (%)	30.5	37.1	37.9	43.5*	37.3
Hypo-HDL cholesterolemia (%)	11.1	17.8	14.7	18.8*	15.6
Dyslipidemia (%)	45.3	56.4*	51.3	57.5*	52.6
Metabolic syndrome (%)	50.5	56.9	53.6	60.2	55.2

Numbers and means with standard deviations, medians with interquartile ranges and frequencies (%) of each variable are shown

Asterisks denote significant differences from the 1st quartile group (* *p* < 0.05; ** *p* < 0.01)

### Comparison of mean levels of each component of metabolic syndrome in the quartile groups for urate in participants with diabetes

Table 5 shows means of each component of metabolic syndrome in the quartile groups for urate in the participants with diabetes. Compared with the 1st quartile, WHtR was significantly higher in the 4th quartile group and log-transformed triglyceride level was significantly higher in the 3rd and 4th quartile groups. Systolic and diastolic blood pressure levels were not significantly different in the 2nd, 3rd and 4th quartile groups compared with the 1st quartile group in multivariate logistic regression analysis. Hemoglobin A1c was significantly lower in the 2nd, 3rd and 4th quartile groups than in the 1st quartile group and tended to be lower with an increase in the quartile [means and standard deviations (%) in univariate analysis: 7.85 ± 1.75 (1st quartile); 7.38 ± 1.39 (2nd quartile); 7.24 ± 1.30 (3rd quartile); 7.11 ± 1.34 (4th quartile)].

**Table 5:** Comparison of means of each variable in quartile groups for urate levels in participants with diabetes

	1st quartile Urate: 0.8–4.7 mg/dl	2nd quartile Urate: 4.8–5.5 mg/dl	3rd quartile Urate: 5.6–6.4 mg/dl	4th quartile Urate: 6.5–12.9 mg/dl
WHtR				
Univariate	0.514 (0.507–0.521)	0.524 (0.515–0.532)	0.522 (0.515–0.529)	0.533 (0.525–0.541)*
Multivariate	0.515 (0.507–0.522)	0.523 (0.516–0.531)	0.522 (0.515–0.530)	0.532 (0.524–0.540)*
Systolic blood pressure (mmHg)				
Univariate	132.6 (130.1–135.2)	134.3 (131.9–136.7)	133.4 (131.2–135.6)**	134.5 (132.1–136.9)**
Multivariate	134.2 (131.9–136.5)	134.4 (132.2–136.6)	133.3 (131.2–135.4)	133.0 (130.6–135.3)
Diastolic blood pressure (mmHg)				
Univariate	79.9 (78.1–81.7)	80.5 (79.0–81.9)	80.5 (79.0–81.9)	83.1 (81.4–84.9)
Multivariate	81.1 (79.6–82.7)	80.7 (79.2–82.2)	80.4 (79.0–81.8)	81.8 (80.2–83.3)
log (triglycerides [mg/dl])				
Univariate	2.02 (1.98–2.06)	2.09 (2.05–2.12)	2.12 (2.08–2.15)*	2.16 (2.12–2.20)**
Multivariate	2.05 (2.01–2.08)	2.09 (2.05–2.12)	2.12 (2.08–2.15)*	2.13 (2.09–2.17)*
HDL cholesterol (mg/dl)				
Univariate	55.9 (53.6–58.2)	51.6 (49.8–53.5)*	51.9 (50.2–53.6)*	52.1 (50.2–54.1)
Multivariate	55.4 (53.6–57.2)	52.0 (50.2–53.7)	51.6 (50.0–53.3)*	52.6 (50.7–54.5)
Hemoglobin A1c (%)				
Univariate	7.85 (7.59–8.10)	7.38 (7.19–7.57)*	7.24 (7.07–7.41)**	7.11 (6.92–7.31)**
Multivariate	7.90 (7.70–8.10)	7.39 (7.20–7.58)**	7.25 (7.06–7.43)**	7.04 (6.84–7.24)**

Means with their 95% confidence intervals for each variable are shown. The range of urate levels in each quartile group is shown in the table. In multivariate analysis, adjusted odds ratios were estimated using age and histories of smoking, alcohol drinking and regular exercise as other explanatory variables. BMI was adjusted in analysis for variables except for WHtR. In addition, a history of medication therapy for hypertension, dyslipidemia or diabetes was used as an explanatory variable in multivariate analysis for systolic and diastolic blood pressure, triglycerides, HDL cholesterol, and hemoglobin A1c

Asterisks denote significant differences from the 1st quartile (* *p* < 0.05; ** *p* < 0.01)

### Comparison of frequencies of metabolic syndrome and each component of metabolic syndrome in the quartile groups for urate in the participants with diabetes

Table 6 shows crude and adjusted odds ratios for metabolic syndrome and each risk factor in the 2nd, 3rd and 4th quartile groups for urate vs. the 1st quartile group in the participants with diabetes. In multivariate logistic regression analysis, odds ratios for metabolic syndrome and any of its components in the 2nd, 3rd and 4th quartile groups vs. the 1st quartile group were not significantly different from the reference level.

**Table 6:** Odds ratios for metabolic syndrome and its components in each quartile group vs. the 1st quartile group for urate in participants with diabetes

	1st quartile Urate: 0.8–4.7 mg/dl	2nd quartile Urate: 4.8–5.5 mg/dl	3rd quartile Urate: 5.6–6.4 mg/dl	4th quartile Urate: 6.5–12.9 mg/dl
Metabolic syndrome				
Crude	1.00	1.29 (0.87–1.93)	1.13 (0.77–1.66)	1.48 (0.99–2.23)
Adjusted	1.00	1.17 (0.68–2.00)	0.82 (0.49–1.37)	0.71 (0.40–1.25)
High WHtR				
Crude	1.00	1.10 (0.73–1.66)	1.16 (0.78–1.74)	1.35 (0.88–2.07)
Adjusted	1.00	1.08 (0.71–1.64)	1.12 (0.75–1.70)	1.27 (0.82–1.97)
Hypertension				
Crude	1.00	1.36 (0.91–2.02)	1.31 (0.89–1.93)	1.97 (1.30–2.98)**
Adjusted	1.00	1.25 (0.80–1.95)	1.13 (0.74–1.74)	1.55 (0.98–2.45)
Hyper-triglyceridemia				
Crude	1.00	1.34 (0.88–2.05)	1.39 (0.92–2.10)	1.76 (1.15–2.68)**
Adjusted	1.00	1.25 (0.81–1.92)	1.22 (0.80–1.89)	1.45 (0.92–2.29)
Hypo-HDL cholesterolemia				
Crude	1.00	1.75 (0.98–3.12)	1.39 (0.78–2.50)	1.87 (1.04–3.34)**
Adjusted	1.00	1.68 (0.92–3.06)	1.55 (0.83–2.87)	1.86 (0.97–3.57)

Odds ratios with their 95% confidence intervals for each variable are shown. The range of uric acid levels in each quartile group is shown in the table. In multivariate analysis, adjusted odds ratios were estimated using age and histories of smoking, alcohol drinking and regular exercise as other explanatory variables. BMI was also adjusted in analysis for variables except for high WHtR. In addition, a history of medication therapy for hypertension or dyslipidemia was used as an explanatory variable in multivariate analysis for hypertension, hyper-triglyceridemia and hypo-HDL cholesterolemia

Asterisks denote significant differences from the reference level of 1.00 (** *p* < 0.01)

### Correlations of urate and related variables with hemoglobin A1c in the overall participants and the participants with diabetes

Table 7 shows the results of univariate and multivariate linear regression analyses for relationships of urate and other variables with hemoglobin A1c. Both in the overall participants and participants with diabetes, urate showed weak but significant inverse correlations with hemoglobin A1c in univariate analysis and multivariate analysis with adjustment for three different sets consisting of other explanation variables (Multivariate-1–3 in Table 7). The correlations between urate and hemoglobin A1c tended to be stronger in the participants with diabetes than in the overall participants.

**Table 7:** Correlations of urate and related variables with hemoglobin A1c in overall participants and participants with diabetes

	Univariate	Multivariate-1	Multivariate-2	Multivariate-3
Overall participants				
Urate	− 0.068 (*p* < 0.001)	− 0.074 (*p* < 0.001)	− 0.124 (*p* < 0.001)	− 0.092 (*p* < 0.001)
Age	–	0.151 (*p* < 0.001)	0.205 (*p* < 0.001)	0.138 (*p* < 0.001)
Therapy for diabetes	–	0.461 (*p* < 0.001)	–	0.460 (*p* < 0.001)
Smoking	–	0.038 (*p* < 0.001)	0.029 (*p* = 0.001)	0.032 (*p* < 0.001)
Alcohol drinking	–	0.054 (*p* < 0.001)	0.076 (*p* < 0.001)	0.065 (*p* < 0.001)
Regular exercise	–	0.004 (*p* = 0.574)	0.022 (*p* = 0.008)	0.007 (*p* = 0.322)
Body mass index	–	0.208 (*p* < 0.001)	0.222 (*p* < 0.001)	0.179 (*p* < 0.001)
Systolic blood pressure	–	–	0.048 (*p* < 0.001)	0.037 (*p* < 0.001)
log (triglycerides)	–	–	0.088 (*p* < 0.001)	0.090 (*p* < 0.001)
HDL cholesterol	–	–	0.002 (*p* = 0.822)	0.015 (*p* = 0.092)
Diabetes				
Urate	− 0.188 (*p* < 0.001)	− 0.222 (*p* < 0.001)	− 0.254 (*p* < 0.001)	− 0.249 (*p* < 0.001)
Age	–	− 0.170 (*p* < 0.001)	− 0.151 (*p* < 0.001)	− 0.140 (*p* < 0.001)
Therapy for diabetes	–	− 0.104 (*p* = 0.003)	–	− 0.082 (*p* = 0.013)
Smoking	–	0.031 (*p* = 0.359)	0.066 (*p* = 0.076)	0.039 (*p* = 0.231)
Alcohol drinking	–	0.020 (*p* = 0.569)	0.051 (*p* = 0.121)	0.077 (*p* = 0.031)
Regular exercise	–	0.009 (*p* = 0.781)	− 0.108 (*p* = 0.002)	0.030 (*p* = 0.352)
Body mass index	–	0.114 (*p* = 0.002)	0.028 (*p* = 0.382)	0.057 (*p* = 0.127)
Systolic blood pressure	–	–	0.091 (*p* = 0.009)	0.081 (*p* = 0.019)
log (triglycerides)	–	–	0.309 (*p* < 0.001)	0.290 (*p* < 0.001)
HDL cholesterol	–	–	0.127 (*p* = 0.001)	0.113 (*p* = 0.004)

Shown are Pearson’s correlation coefficients in univariate analysis and standardized partial regression coefficients in multivariate analysis. Multivariate analysis was performed under three conditions (Multivariate 1–3) using different sets of explanatory variables

The *p* value for each correlation coefficient is shown in parenthesis

## Discussion

The results of ANCOVA and logistic regression analysis using the quartile groups for urate showed an inverse association between urate and glycemic status in overall participants. This association was confirmed by the results of linear regression analysis and agrees with the finding that the proportion of participants with a history of medication therapy for diabetes tended to be smaller with an increase in the quartile for urate. An inverse association between urate and glycemic status was also found in ANCOVA using data for the participants with diabetes. Therefore, serum urate is inversely associated with glycemic status both in a general population and in persons with diabetes. The present study is the first study demonstrating inverse associations between serum urate level and glycemic status in a general population and patients with diabetes in the same cohort.

Results of previous studies regarding the relationships of urate with glycemic status and diabetes were conflicting [3–13]. There were two previous studies [2, 3] with study designs similar to the study design of the present study. In those studies, prevalences of diabetes were compared in 4 groups of different blood urate levels, and the results of those two studies were controversial: The prevalence of diabetes in the study by Oda et al. [3] using a database of Japanese citizens and that in the study by Choi et al. [2] using a database of US citizens tended to be lower and higher, respectively, with an increase in urate level. The findings of the present study using a cohort of Japanese agree with the findings of the study by Oda et al. Therefore, one possible explanation for the above conflicting results is the difference in ethnicity. Regarding the relationship between urate and glycemic status in patients with diabetes, an inverse association between serum urate and hemoglobin A1c was found in a study in which a database of Chinese participants were used [13]. This finding agrees with that of the present study, although a positive association between urate and hemoglobin A1c in non-diabetic participants was found in the above study in China [13]. Therefore, further studies are needed to clarify whether ethnicity affects the relationships of urate with glycemic status and diabetes.

On the other hand, in the present study, urate was positively associated with metabolic syndrome and its components, except for diabetes, including visceral obesity, hypertension and dyslipidemia such as hypertriglyceridemia and hypo-HDL cholesterolemia. A dose–response relationship was found between serum urate level and prevalence of metabolic syndrome in non-diabetic participants. These findings agree with the findings of previous studies [2–4] and are reasonable since there are similar lifestyle-related backgrounds in hyperuricemia and the components of metabolic syndrome [17].

Although the reason for the inverse association between urate level and glycemic status is unknown, there is a possibility of involvement of insulin in changes in serum urate level in patients with diabetes: Insulin enhances renal tubular reabsorption of urate [18, 19], and thus in patients with diabetes, insulin deficiency increases urinary excretion of urate, resulting in a decrease in blood urate level. This mechanism may explain the inverse association between serum urate level and glycemic status in patients with diabetes and the bell-shaped relationship between glycemic status and blood urate level: In persons with prediabetes, hyper-insulin status causes a reduction of urinary urate excretion, resulting in higher blood urate level than in persons with normo-glycemic status [8, 9]. Thus, the relationship between hemoglobin A1c and urate in those with diabetes might be just a reflection of reverse causation in which those with diabetes have increased urinary output and clearance of uric acid such that those with high hemoglobin A1c have less serum urate. Another possible explanation for the inverse association between urate and diabetes is an antioxidant property of urate in the blood [20, 21] since oxidative stress is known to be involved in the pathogenesis of diabetes through development of insulin resistance and deterioration of insulin secretion [22]. Thus, low urate in the blood is hypothesized to induce impaired glucose tolerance through elevating oxidative stress level. Moreover, in a recent retrospective longitudinal study, serum urate was inversely associated with development of metabolic syndrome [23], and the authors of that report speculated that there is an anti-oxidant effect of urate on the pathogenesis of metabolic syndrome. Further studies are needed to determine whether urate contributes to oxidative stress level in the blood and is involved in diabetologenesis.

For the above reasons, more participants who showed low urate levels are thought to have been included in the participants with more severe diabetes. This may result in weaker associations of urate level with metabolic syndrome and its components (except for diabetes) in participants with diabetes than in the overall participants: High urate level was positively associated with visceral obesity, hypertension, dyslipidemia (high triglycerides and low HDL cholesterol) and metabolic syndrome, and these associations were less prominent in the diabetes group than in the overall participants. On the other hand, the correlation between urate and hemoglobin A1c was stronger in the participants with diabetes than in the overall participants. This may be explained by a wider range of insulin status, which influences serum urate levels through renal excretion, in the diabetes participants than in the overall participants.

There are limitations of this study. First, the participants of this study were Japanese and, as discussed above, there is a possibility of an ethnicity-related difference in the relationship between urate and glycemic status. To confirm this hypothesis, the relations of urate level with glycemic status and frequency of diabetes should be compared among groups of participants with different ethnicities in future studies with similar designs. Second, the participants of the present study were all men; however, there is a possibility of a gender-related difference in the relationship between urate and glycemic status. In fact, mean urate level in blood is lower in women than in men [24]. In a previous study using a cohort in Japan, there was an inverse association between serum urate level and prevalence of diabetes in men, while no significant relationship between them was found in women [3]. Analysis of data for women (n = 4884) was also tried in this study. However, the prevalence of diabetes was much lower in women (2.1%) than in men (6.4%) and the number of female subjects with diabetes was too small to investigate the relationship between serum urate level and diabetes. Third, insulin affects clearance of urate in the kidney and it would be interesting to analyze insulin levels in relation to blood urate and hemoglobin A1 levels. However, information on insulin level was not included in the database used and was not available in this study. Fourth, in multivariate analyses, lifestyle-related factors such as smoking, drinking and regular exercise were included in variables for adjustment. Alcohol drinking was evaluated by frequency of drinking. However, it is known that urate level is affected by the kind of alcohol beverages [25], for which information was not available in the database used in this study. Fifth, the anti-dyslipidemic drugs such as atorvastatin and simvastatin, but not the other statins, have been shown to lower serum urate levels [26]. In addition, fenofibrate, but not bezafibrate, has been shown to be effective in reducing serum urate levels [27]. However, information on the names of anti-dyslipidemic drugs was not included in the database used in this study. Finally, this study is cross-sectional in its design, and thus no causality can be discussed here. In a prospective study using a cohort in Japan, serum urate level was associated positively with impaired glucose tolerance and diabetes [28]. Further studies are also needed to elucidate the reasons for the controversy in findings of prospective studies and cross-sectional studies on the relation between urate and diabetes.

## Conclusion

Blood urate level is inversely associated with glycemic status both in a general population and in persons with diabetes, and all of the components of metabolic syndrome except for blood sugar are positively associated with urate level. The significance of the inverse association between uric acid and glycemic status in diabetes remains to be determined.

## Data Availability

The datasets used and/or analyzed during the current study are available from the corresponding author on reasonable request.

## Abbreviations

ANCOVA: Analysis of covariance
ANOVA: Analysis of variance
BMI: Body-mass index
HDL: High-density lipoprotein
IDF: The International Diabetes Federation
JDS: The Japan Diabetes Society
NGSP: National Glycohemoglobin Standardization Program
WHtR: Waist-to-height ratio
